# Tumor Targeting and Drug Delivery by Anthrax Toxin

**DOI:** 10.3390/toxins8070197

**Published:** 2016-07-01

**Authors:** Christopher Bachran, Stephen H. Leppla

**Affiliations:** 1 BioMed X Innovation Center, Heidelberg 69120, Germany; 2 Laboratory of Parasitic Diseases, National Institute of Allergy and Infectious Diseases, National Institutes of Health, Bethesda, MD 20892, USA; sleppla@niaid.nih.gov

**Keywords:** drug delivery, immunotoxin, targeted toxin, cancer, tumor therapies

## Abstract

Anthrax toxin is a potent tripartite protein toxin from *Bacillus anthracis*. It is one of the two virulence factors and causes the disease anthrax. The receptor-binding component of the toxin, protective antigen, needs to be cleaved by furin-like proteases to be activated and to deliver the enzymatic moieties lethal factor and edema factor to the cytosol of cells. Alteration of the protease cleavage site allows the activation of the toxin selectively in response to the presence of tumor-associated proteases. This initial idea of re-targeting anthrax toxin to tumor cells was further elaborated in recent years and resulted in the design of many modifications of anthrax toxin, which resulted in successful tumor therapy in animal models. These modifications include the combination of different toxin variants that require activation by two different tumor-associated proteases for increased specificity of toxin activation. The anthrax toxin system has proved to be a versatile system for drug delivery of several enzymatic moieties into cells. This highly efficient delivery system has recently been further modified by introducing ubiquitin as a cytosolic cleavage site into lethal factor fusion proteins. This review article describes the latest developments in this field of tumor targeting and drug delivery.

## 1. Introduction

*Bacillus anthracis* is a Gram-positive, endospore-forming, rod-shaped bacterium which is the causative agent of the disease anthrax. The spores are highly resistant to harsh environmental conditions like heat, ultraviolet light, radiation, pressure, or chemical agents [1]. Spores can survive for decades in soil until they are inhaled or ingested by animals, such as domestic livestock [2]. Once back in a favorable environment they germinate and grow rapidly. The spores are important in the early stages of infection [3]. *B. anthracis* produces two factors which are essential for full virulence. One is the poly-γ-d-glutamic acid capsule, which is antiphagocytic and protects the bacteria from immune surveillance and allows its growth in the host without hindrance [4]. The genes relevant for synthesis of the poly-γ-d-glutamic acid capsule reside on the *B. anthracis* plasmid pXO2. The second virulence factor is the secreted anthrax toxin [5]. Anthrax toxin (AT) consists of three proteins: protective antigen (PA, 83 kDa), lethal factor (LF, 90 kDa) and edema factor (EF, 89 kDa). These three polypeptides are encoded by the *B. anthracis* plasmid pXO1 and form bipartite combinations. The combination of LF and PA generates lethal toxin (LT), while EF combined with PA comprises edema toxin (ET). LT and ET cause anthrax and the disease-related symptoms. In mouse experiments, the lethality of both LT and ET has been shown [6], while the three toxin components (PA, LF, and EF) are not individually toxic [1]. The main role of the poly-γ-d-glutamic acid capsule is to facilitate initiation of an infection, while the symptoms of anthrax disease are due to septicemia, which is the result of the toxin production (reviewed in [3]). PA, LF, and EF have been studied in great detail and this has resulted in detailed knowledge of their molecular mechanisms of action.

**Figure 1:**
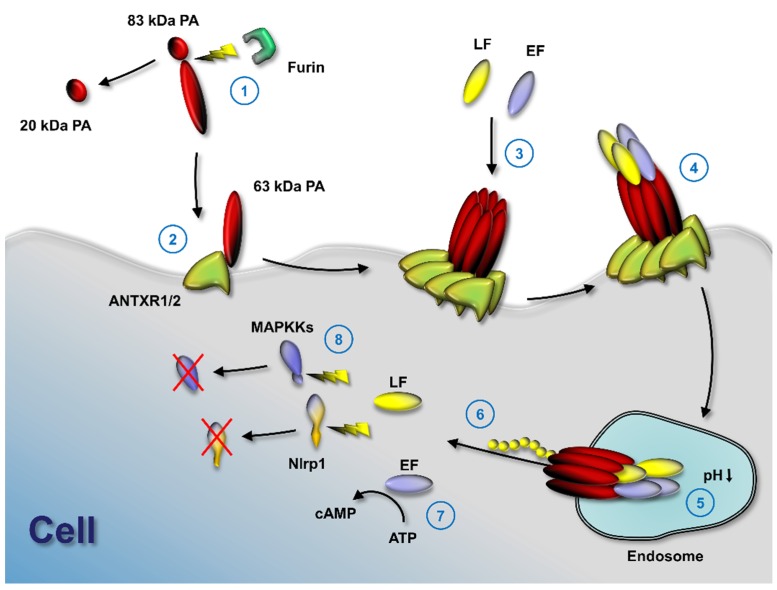
Anthrax toxin activation mechanism. Anthrax toxin protective antigen (PA) is cleaved by furin (1) to enable binding to either of its two known cell surface receptors (2), tumor endothelial marker 8 (TEM8 or ANTXR1) or capillary morphogenesis gene 2 (CMG2 or ANTXR2). PA oligomerizes (3) and the two effector molecules of anthrax toxin, lethal factor (LF) and edema factor (EF), bind to the PA oligomer. If PA forms an octamer, up to four effector molecules can bind at the intersection of PA molecules and internalize by receptor-mediated endocytosis (4); acidification in endosomes results in pore formation by PA (5); LF and EF partly unfold and the unfolded *N*-terminus is pulled through the pore by a ratchet mechanism. Unfolded LF and EF refold in the cytosol (6) and exhibit their enzymatic functions. EF catalyzes the formation of cytosolic cAMP (7) and LF cleaves several mitogen-activated protein kinase kinases, as well as Nlrp1 (8).

LF and EF can only access host cells through delivery by PA (Figure 1). PA binds to either of its two known cell surface receptors, tumor endothelial marker 8 (TEM8 or ANTXR1) or capillary morphogenesis gene 2 (CMG2 or ANTXR2). Results indicate that CMG2 is the major receptor responsible for in vivo toxicity in mice [7,8]. Cell surface binding of PA results in its oligomerization, which generates binding sites for LF and EF [9,10]. In recent years, the molecular mechanisms of the anthrax toxin intoxication process has been studied in great detail and is understood very well (reviewed in [11]). The first step of the uptake mechanism is the binding of PA to TEM8 or CMG2. Both receptors are transmembrane proteins and ubiquitously expressed. TEM8 has also been described to be overexpressed in breast cancer [12], gall bladder carcinomas [13], and prostate cancer [14]. CMG2 might play a role in parturition as the knock-out of CMG2 in mice resulted in the inability of pregnant mice to give birth [15]. A deposition of collagen was found in the myometrium of the mice, resulting in loss of smooth muscles. In human patients, mutations of CMG2 are connected to two autosomal recessive disorders, Juvenile Hyaline Fibromatosis and Infantile Systemic Hyalinosis. Both diseases are characterized by excess hyaline material deposition in connective tissues. The role of CMG2 and TEM8 in healthy individuals remains unclear. In a valuable study, Liu et al. described the role of tissue-specific CMG2-knockouts in mice and the effect of LT or ET in these mice [6]. As a result, LT acts specifically on cardiomyocytes and vascular smooth muscle cells expressing CMG2, while ET acts mainly on CMG2-positive hepatocytes. According to this study, the role of TEM8 as an AT receptor is negligible for intoxication by LT and ET in mice.

After binding to CMG2 or TEM8, the 83-kDa PA protein is cleaved by furin or furin-like proteases to yield a 63-kDa protein which remains bound to the receptor while the 20-kDa fragment is released [16]. The cleavage of PA initiates oligomerization to form a ring-shaped heptamer or octamer [17,18]. The octamer of PA seems to be more prevalent under physiological conditions [19]. The octamer is more stable (and, thus, more cytotoxic on macrophages in combination with LF) in serum. LF and EF can only bind to sites that are created by the formation of the PA oligomer since the site spans two PA molecules. The oligomerization also triggers receptor-mediated endocytosis of the complex by clathrin-dependent internalization into endosomes. However, Kintzer et al. report octameric PA complexes bound to LF in serum [19]. Thus, complex formation may be independent of cell binding in some circumstances. The acidification of the endosome causes the PA oligomer (also called the PA pre-pore) to insert into the endosome membrane and to form a pore through which LF and EF translocate into the cytosol [20]. LF and EF need to unfold to be delivered through the PA pore into the cytosol of cells. The structure of the PA pore has been solved at a 2.9 Å resolution recently by cryo-electron microscopy [21]. The structure shows the narrow pore and underlines the necessity for LF and EF to unfold to pass through the pore. The structural constraints of the PA pore limit translocation to only those proteins able to completely unfold. PA pore formation triggers partial unfolding of the *N*-terminus of LF or EF and the unfolded *N*-terminus binds to the amphipathic α clamp on the surface of the PA pore [22]. The translocation of the proteins requires a proton gradient and is probably following a Brownian ratchet mechanism [23]. The structural analysis of the PA pore supports the proposed Brownian ratchet mechanism [21]. The primary amino acid sequence of the *N*-terminus of LF and EF is essential for efficient translocation. In addition, studies have shown the requirement of the chaperone GRP78 for efficient translocation into cells [24]. Chaperones might help with the correct folding of the protein toxins in the cytosol and facilitate translocation. However, another study indicated that the chaperones cyclophilin A and heat shock protein 90 facilitate the translocation of a LF fusion protein, but not of LF [25].

LF and EF are enzymes with completely different functions. EF is a calmodulin-dependent adenylate cyclase [26] which increases the cAMP concentration in cells and helps spreading *B. anthracis* in the host by affecting signaling pathways and modulating immunologic responses. ET injected in high doses is lethal in mice and induces hemorrhaging lesions in many organs accompanied by both hypotension and bradycardia [27]. However, while ET manifests massive immunomodulatory effects in immune cells, such as macrophages and B cells [28], it induces no acute cytotoxic effect on a number of cell types, such as dendritic cells, T-cells, macrophages, neutrophils, and human microvascular endothelial cells [29,30,31,32,33]. LF is a zinc metalloproteinase that cleaves mitogen-activated protein kinase kinases (MAPKKs) in their *N*-terminal regions [34] and Nlrp1 [35]. The cleavage of Nlrp1 by LT causes toxin-induced inflammasome activation, IL-1β release, and pyroptosis of macrophages in certain strains of mice and rats [35]. The cleavage of MAPKKs disrupts several signaling pathways, including the ERK1/2, JNK/SAPK, and p38 pathways, all of which are important in numerous cellular functions such as proliferation and cell cycle regulation, but also immune modulation and survival against toxic insults [34,36,37,38]. The concerted action of both toxins is believed to help overcome the innate immune response (reviewed in [3,6]).

The three components of AT are individually non-toxic, and the PA component must be proteolytically activated prior to cell intake. These unique features render anthrax toxin attractive for tumor therapy. By introducing small modifications to restrict its action to specific tumor cells, it becomes a potential treatment in cancer therapy. One successful approach involves mutating PA to make it susceptible to tumor-associated proteases instead of to the ubiquitously-expressed furin protease [39,40]. The unique features of AT and the variety of possible modifications that restrict it to specific cell types and the possibility of being an effective delivery system show that AT is a promising tool in targeted tumor therapy and other fields of biomedicine.

## 2. Tumor-Selective Activation of Protective Antigen and Tumor-Selective Formation of Protective Antigen Octamer

A number of proteases are highly expressed in tumors and, thus, might be used for tumor-selective activation of pro-drugs. This idea is established in the field of tumor therapeutics and studied for many different proteases and drugs (reviewed in [41]). In a related approach, the requirement for the proteolytic activation of PA has been used for the tumor-selective activation of PA and, thus, the tumor-selective delivery of LT to tumor cells. The first study describing a mutated PA with changed protease sensitivity was published in 2000 by Liu et al. The furin-sensitive site in PA was mutated to instead be cleaved by matrix-metalloproteases (MMPs) [40]. In a subsequent study, the protease sensitivity was changed for urokinase plasminogen activator (uPa)-activation [39]. These PA variants greatly increased the specificity for certain tumor cells lines and allowed for subsequent preclinical testing of modified anthrax toxin in the context of tumor therapies. A further improvement of specificity of modified anthrax toxin was achieved by designing PA variants that required simultaneous activation by both uPa and MMPs to form functional oligomers [42]. The details on these modified anthrax toxins are reviewed in detail by other authors in this volume and we refer you to the review by Liu et al. for the details on protease-specific activation of anthrax toxin.

This idea of increased target specificity was further improved by engineering PA variants that can only form octamers after activation by both of the tumor-selective proteases, uPa and MMPs and, thus, achieved a safe dual-activity dependent delivery system [43]. The recognition that PA can form functional octamers came only in 2009 [18], so the potential of the octamer to become a useful drug delivery tool has not yet been fully investigated. Phillips et al. used the concept of PA octamers and introduced the D512K mutation into PA (PA-DK), preventing the formation of PA oligomers [44]. Based on this knock-out mutation, the authors generated a PA library and identified other mutations overcoming the D512K mutation to form PA oligomers despite the initial mutation. One of the characterized mutations was named PA-GN. Upon combination of these two PA variants, PA-DK and PA-GN, with either of the aforementioned uPa or MMP cleavage sites for PA activation, PA oligomerization and LF uptake into cells was made dependent on dual protease activation. This resulted in impressive tumor growth inhibition on A549 xenografts and improved the safety over the previous dual-protease activation system. Thus, it should be possible to use the gained knowledge on PA octamers and protease-specific activation to design safer variants of tumor-specific AT for future preclinical and clinical testing.

## 3. Cellular Delivery of Fusion Proteins

In 1992 Arora et al. described, for the first time, the delivery of other enzymes into cells by PA and LF. The work presented described genetic fusions of LF and fragments of *Pseudomonas* exotoxin A (PE) [45]. PE is an ADP-ribosylating exotoxin from *Pseudomonas aeruginosa*, a potent inhibitor of protein synthesis and inducer of apoptosis. The toxin consists of a receptor-binding domain (domain I), a translocation domain (domain II), and a catalytic domain (domain III). The fusions with LF contained either PE domains II and III (PE38), lacking the toxin’s own targeting domain, or only PE domain III (PEIII), the catalytic domain. The advantage of fusing LF with this enzyme is an easy and fast detection of PE activity in cells, either by detecting ADP-ribosylation on PE’s cellular target, eukaryotic elongation factor 2, or by determining protein synthesis inhibition and decreased cell survival upon protein synthesis inhibition and apoptosis induction. It is important that proteins fused to LF completely unfold in order to pass through the PA pore. While this discovery at the time was mainly meant to help understanding the molecular mechanism of AT, it opened a whole new chapter for using AT as a drug delivery system and started a series of many studies on AT for the use in tumor therapies. The initial study was performed on full length LF and in order to understand the essential part of LF for the delivery of other proteins into cells, a further study investigated the cellular uptake of different fragments of LF genetically-fused to PEIII [46]. These studies showed that only the *N*-terminal domain of LF (LFn, amino acids 1–254) is required for the transport of polypeptides into cells. The *N*-terminus of LF is important for the efficient uptake and consists of an amphipathic sequence with many lysine residues. Later studies identified polycationic peptides to be sufficient for PA-mediated uptake of enzymes into cells [47]. The positively-charged tag needs to be on the *N*-terminus of the enzyme to be delivered and lysine residues were more effective than arginine or histidine residues [48]. However, the efficiency is usually much lower compared to LFn. Based on the success of LFn genetic fusions, a series of studies was initiated to evaluate the potential of PA/LFn as a drug delivery system in molecular medicine. In parallel to the discovery that LFn is sufficient for the delivery of PE to the cytosol of PA-binding cells, the successful delivery was shown for additional protein toxins. Both diphtheria toxin A chain (DTA, the catalytic domain of diphtheria toxin) and Shiga toxin catalytic domain were successfully delivered to the cytosol of CHO cells [49,50]. DTA is an ADP-ribosylating enzyme with the same mechanism as PEIII. Shiga toxin is a ribosome-inactivating protein (an RNA *N*-glycosidase), which removes a specific adenine from 28S-rRNA, resulting in protein synthesis inhibition. Many of these early experiments were performed on CHO-K1 cells, which are sensitive to the combination of wild-type PA and LFn-PEIII. While PA, and also tumor-selective PA variants cleaved by other proteases alone (without LF or cytotoxic LFn fusion proteins), are not cytotoxic to a variety of cell lines, it was reported that recombinant CHO cell lines overexpressing TEM8 (CHO-TEM8) are sensitive towards PA without any LF or LFn fusion protein [51]. One study investigated effects of PA on tumor growth without the combination with effector molecules due to the expression of CMG2 and TEM8 during angiogenesis. Rogers et al. analyzed the effect of PA on growth factor-induced angiogenesis and identified a PA variant with increased angiogenesis inhibitory effects and tumor growth inhibitory effects in vivo [52]. This PA variant PASSSR did not undergo endocytosis and stayed bound to its receptors on the cell surface. Three- to five-fold higher concentrations of PASSSR were used in comparison to other studies.

The combination of LFn-PEIII was subsequently used in a number of publications to efficiently target and eliminate tumor cells both in vitro and in vivo with different PA variants for targeting a great variety of different tumor types [39,40,42,43,53,54,55,56,57,58,59]. PA variants that selectively target tumor cells successfully delivered the highly-potent LFn-PEIII to tumor cells, resulting in potent tumor growth inhibition in many studies. In A549 tumor xenografts [43] and non-small cell lung cancer models [58] tumor regression was observed in all treated mice with lasting regression in many cases (up to 30%).

While many tumor cells are rather resistant to LF, protein synthesis inhibition and induction of apoptosis by PE affects the vast majority of tumor cells. A number of melanoma cell lines are sensitive to LF and this was demonstrated in several studies. In a study of 25 melanoma cell lines, Alfano et al. described the high activity of LF on cell lines having the activating V600E B-RAF mutation, while other melanoma cell lines were resistant [60]. This was confirmed in another study testing additional cell lines [61]. Even in a sub-cutaneous xenograft melanoma model, the combination of PA and LF resulted in partial and complete remissions [62]. The combination of MMP-activated PA with LF obtained excellent results also in melanoma xenografts, and lung and colon carcinoma xenografts irrespective of the B-RAF status [63]. This was attributed to the detrimental effect of the toxin combination on the tumor vasculature and angiogenic processes. This was reported at the same time by Huang et al. [64]. In the case of soft tissue sarcomas, AT acts directly on tumor cells and due to the dependence of fibrosarcoma on MAPKK, in vivo treatment of mice with AT resulted in reduced fibrosarcoma growth and reduced neovascularization [65]. Rouleau et al. reported efficacy of AT in a neuroblastoma mouse model, probably also due to targeting of the tumor vasculature, since no correlation between target receptors and PA sensitivity was reported in vitro [66]. In an alternative approach, Zhuo et al. delivered full-length LF encoded in a viral vector to human A549 lung carcinoma cells and expressed the protein toxin under the control of the tumor-specific human telomerase reverse transcriptase promoter [67]. The high expression of LF resulted in cleavage of MAPKKs, apoptosis induction and moderate growth inhibition of A549 cells due to their apparent sensitivity to MAPKK pathway interference. The resistance of many tumor cells towards the inhibition by LF can be overcome by combinations with chemotherapeutics. Wein et al. indicated that B16-BL6 melanoma cells resistant to LF are affected by a combined application of uPa-activated PA/LF and paclitaxel (microtubule degradation inhibitor) in vivo [68]. The combination of paclitaxel and uPa-activated PA/LF resulted in additive (not synergistic) effects on tumor growth inhibition. The effect was not observed in vitro, since LF affects the tumor vasculature in this tumor model. This positive result of the combination supports the idea that combinations of tumor-selective AT with chemotherapeutics might help target otherwise resistant tumors due to independent mechanisms of action. On the other hand, development of LFn genetic fusions to PE might help overcome this obstacle, since only very few tumor cell lines show resistance to PE [69]. One example of PE-resistant small cell lung carcinoma cell lines was described by Mattoo et al. and was overcome by the combination with BH-3 only peptide mimetic ABT-263 [70]. Despite the inhibition of protein synthesis, apoptosis was not induced in those cells upon PE treatment. Reduced levels of Mcl-1 due to the inhibition by ABT-263 circumvented the resistance of the cancer cell lines towards PE. A further advantage of PE in addition to its high potency is the intense published work on reducing the immunogenicity of PE. In recent years the group of Ira Pastan developed several immunotoxins based on PE by using different antibodies or antibody fragments and expressed them as fusion proteins with PE fragments PE38 or PEIII. These fusion proteins were studied in a number of clinical trials and resulted in the identification of T cell epitopes [71] and B cell epitopes [72]. The careful mutations of epitopes on the surface of PE38 retained the enzymatic activity of the enzyme, while preventing immune responses upon use of the fusion proteins. In addition, it was shown by Mossoba that the combined application of pentostatin and cyclophosphamide greatly reduces immunogenicity of a PE38-containing immunotoxin in mice [73]. The immunotoxin tested contained a disulfide-stabilized Fv targeting mesothelin and PE38. The drug combination was administered starting six days prior to immunotoxin application and resulted in a nearly complete depletion of B cells and T cells when measured at the end of the experiment after three weekly injections of the immunotoxin. As a result, the mice produced no detectable amounts of specific antibodies. A drastic depletion of CD4+ and CD8+ T cells, as well as B cells, was reported. However, myeloid cells (CD11b+ and Gr-1+) cells were also affected. Based on current knowledge, the CD11b+ and Gr-1+ myeloid-derived murine suppressor cells play an important role in suppressing T cells in their anti-tumor activity. This might even yield a positive additional effect of the potentially unwanted removal of T cells. An additional treatment regimen with rapamycin co-administration resulted in a weaker reduction of T and B cell deletion. In a subsequent study on ten mesothelioma patients, the combination of pentostatin and cyclophosphamide clearly reduced the levels of CD4+ and CD8+ T cells as well as CD19+ B cells in every patient (measured after one 30-day cycle of immunosuppression and immunotoxin administration) [74]. Platelets and neutrophils were constant in most patients after the treatment. Antibody responses were delayed and neutralizing antibodies were detected in most patients only after the second treatment cycle. The treatment regimen resulted in partial responses in three out of ten patients, a promising result for patients with advanced mesothelioma. Thus, the combination of highly effective pentostatin and cyclophosphamide might reduce the immunogenicity of AT components (e.g., PA with LFn-PEIII) sufficiently to allow for the repeated application of the proteins without raising strong immune responses.

Intracellular stability might play a role for all the discussed fusion proteins of LFn, since the proteins need to be present and active in the cytosol of targeted tumor cells to initiate cell killing. Gupta et al. discovered that the *N*-end rule is important for LFn stability and LFn fusion protein cytotoxicity. The *N*-end rule fully applies to LFn fusion proteins. Thus, LFn fusion proteins with stabilizing amino acids at their *N*-terminus have significantly increased cytotoxicity [75,76]. Degradation of the cytosolic proteins occurs via ubiquitination. In an attempt to further stabilize LFn fusion proteins, Bachran et al. modified proteins by reductive methylation in order to mask lysine residues on the protein surface. The procedure decreased ubiquitination of LFn-PEIII and drastically reduced the immunogenicity of the fusion proteins. In another study, a ubiquitin linker was introduced between LFn and PEIII to increase the persistence of PEIII in the cytosol and, thus, the potency of PEIII after delivery to tumor cells [77]. The ubiquitin linker was efficiently cleaved in the cytosol and PEIII was enriched due to its inherent cytosolic stability. This modification of the well-studied LFn-PEIII helped for the development of an effective anti-tumor drug.

In addition to the well-studied and well-described PEIII, some other protein toxins have been used in combination with LFn to be delivered specifically to tumor cells. Both LFn-DTA and LFn-ricin toxin A chain were combined with a mutant PA unable to bind to CMG2 or TEM8 (mPA), and fused to an affibody against human HER2 (mPA-ZHER2) for targeting of HER2-positive human cancer cell lines [78]. DTA has been described earlier and is very similar to PEIII. Ricin toxin A chain is the catalytic domain of the ribosome-inactivating protein ricin, a potent inhibitor of protein biosynthesis similar to the aforementioned Shiga toxin. Both LFn fusion proteins were highly potent inhibitors of protein synthesis and cell survival. LFn-DTA achieved 50% cell killing with concentrations of 0.1 pM on high HER2-expressing BT474 cells. Yet another example is cytolethal distending toxin. Bachran et al. introduced the fusion protein LFn-cytolethal distending toxin B (CdtB), containing the catalytic B subunit of the tripartite cytolethal distending toxin [79]. CdtB is mainly regarded as a double-strand break-inducing deoxyribonuclease enzyme. Its fusion to LFn and combination with PA resulted in increased DNA damage, cell cycle arrest, and eventually cell death after 72 h (in contrast PEIII induces cell death commonly after 24–48 h toxin exposure). The potent combination with a tumor-selective MMP-activated PA variant obtained an impressive 90% cure rate in a Lewis lung carcinoma mouse model.

LFn-DTA has been used for PA pore translocation experiments by Rabideau et al. [80]. LFn-DTA was enzymatically conjugated to various non-canonical peptides (a 10-amino acid peptide with non-canonical modifications of the side chains) and translocation was not reduced. Conjugation of cyclic peptides of similar length drastically reduced translocation due to steric hindrance and the inability of the cyclic peptide to unfold. The cytostatic drugs doxorubicin and monomethyl auristatin F were also successfully translocated after fusion to LFn-DTA, while the bulkier drug docetaxel was less efficiently translocated. Even antibody mimetics (monobodies derived from the tenth type III domain of human fibronectin, affibodies derived from the immunoglobulin binding protein A, DARPins based on ankyrin repeat modules, and the B1 domain of protein G) were successfully translocated after fusion to LFn-DTA or just LFn [81]. None of the four antibody mimetics reduced translocation efficiency for LFn-DTA, indicating that the fusion proteins completely unfolded. Furthermore, the LFn fusions delivered the antibody mimetics to the cytosol where the proteins successfully refolded and bound to their intracellular targets such as Bcr-Abl kinase or rapidly accelerated fibrosarcoma-1 (Raf-1) kinase. The work by the group of Pentelute describes several examples of successful delivery of molecular moieties not described earlier (i.e., small molecules and antibody mimetics). As long as steric limitations of the PA pore are passed and unfolding/refolding for proteins can be achieved, LFn/PA efficiently delivers various cargos to cells.

LF and LFn have also been fused to other proteins and molecules not related to tumor therapies. In an early experiment, LF was genetically fused to the tetanus toxin light chain and the combination of the fusion protein with PA resulted in successful uptake of the tetanus toxin light chain [82]. The fusion protein induced cytotoxicity in mouse macrophage cell lines and CHO cells. Four further LFn genetic fusion proteins to enzymes or proteins have been reported in the literature; LFn was genetically fused with gp120 in order to deliver gp120 to major histocompatibility complex class I antigen-presenting cells for the development of novel T cell vaccines [83]. The delivery was mediated by PA and resulted in the successful presentation of a detectable epitope on targeted cells. LFn was also used to deliver B-cell lymphoma-extra large (Bcl-XL) into neurons to prevent neurodegenerative diseases and trauma [84]. The genetic fusion of LFn and Bcl-XL successfully inhibited apoptosis in cerebellar granule cells and macrophages Furthermore, LFn-flagellin (*Legionella pneumophila* flagellin) was efficiently delivered to the cytosol of mouse macrophages by PA [85] and flagellin activated the inflammasome in the macrophages. LFn was also combined with β-lactamase to generate a screening and visualization system based on AT [86,87,88,89]. The combination of PA and LFn-β-lactamase has been successfully used to characterize the protease specificity for uPA-activated and MMP-activated PA and to identify inhibitors for the uptake of AT by utilizing a membrane-permeable β-lactamase substrate.

Furthermore, LFn genetic fusions to peptides have been described for vaccination purposes. LFn was fused to a short nine-amino acid cytotoxic T cell epitope from an intracellular pathogen, *Listeria monocytogenes*, and achieved T cell activation upon treatment of mice with PA and the LFn fusion [90]. A very similar study was performed with a fusion of LFn to an eight-amino acid epitope of ovalbumin [91,92]. Eventually, even two epitopes were fused to LFn (an epitope of nucleoprotein of lymphocytic choriomeningitis virus and an epitope of listeriolysin O protein) and mounted a T cell response in mice and reduced the virus load in vaccinated and lymphocytic choriomeningitis virus-infected mice [93].

Cellular protein and peptide delivery by PA and LFn is a robust and efficient system used for many approaches and with success in many anti-tumor studies. Efficient targeting of specific cell populations is key to a successful utilization of AT in anti-tumor therapy and biomedicine.

## 4. Retargeting of PA

One way to reprogram the targeting of PA to its receptors TEM8 and CMG2 is to introduce mutations that enable preferably binding to either of the two receptors. This was achieved by Chen et al. by using PA N657Q with lower cytotoxicity towards TEM8-expressing cells while CMG2-expressing cells were still affected as with wild-type PA [94]. Furthermore, PA R659S/M662R had enhanced specificity towards TEM8-expressing cells, making this PA variant another interesting tool for targeted therapies. In a recent publication, Chen et al. described two further mutants of PA (PA I656Q and PA I656V) with decreased affinity towards TEM8 but with maintained high affinity for CMG2 [95].

The first approach to target PA to specific cells was published in 1998 by Varughese et al. [54]. The amino acids 410–419 of the human p62 (c-myc) epitope were fused to the C-terminus of PA to redirect PA to a c-Myc-specific hybridoma cell line. To prevent undesired binding of PA to its natural receptors, the inactive inhibitor PA SNKE-DeltaFF was added to the cells. In combination with LF or cytotoxic LFn-PEIII, cellular toxicity was specific for PA-c-myc. A more elegant approach was published in 2012 by McCluskey et al. [96]. The authors utilized a mutant PA that is unable to bind either TEM8 or CMG2 based on two point mutations, N682A and D683A [97]. This mutant PA (mPA) was fused C-terminally to human epidermal growth factor (mPA-EGF) [96]. The combination of mPA-EGF with the genetic fusion LFn-DTA resulted in high protein synthesis inhibition (IC_50_ 0.01 nM LFn-DTA) on epidermal growth factor receptor-positive human A431 tumor cells while protein synthesis in receptor-negative CHO cells was not affected at concentrations of up to 10 nM LFn-DTA. The combination of LFn-DTA with mPA-EGF was even 10-fold more efficient than with wild-type PA. Human A431 epidermoid carcinoma cells express very high levels of epidermal growth factor receptor, which may explain this result. Additional experiments on cell lines with different amounts of target receptor and TEM8 or CMG2 might show the difference in a clearer way. The successful retargeting of PA was subsequently demonstrated for a different receptor, HER2 [78]. In this study, the authors showed specific binding of mPA fused to the affibody ZHER2 (mPA-ZHER2) to HER2-positive breast cancer cell lines. The 58-amino acid long affibody ZHER2 is a HER2-binding peptide based on protein A from *Staphylococcus aureus* [98]. Using an artificial ligand for HER2 is the only option to target HER2 directly, since no natural ligand has been described. The study was performed on human A431, BT-474, MDA-MB-468, SKBR3, MDA-MB-231, and JIMT-1 cells with varying levels of HER2 expression. mPA-ZHER2 was combined with either LFn-DTA or LFn-RTA and achieved strong inhibition of protein synthesis and high cytotoxicities on HER2-positive cells, while HER2-negative cells were not affected. This was very elegantly demonstrated in co-culture of HER2-positive and -negative cell lines with a resulting eradication of HER2-positive cells. In a subsequent publication the same affibody ZHER2 and the HER2-targeting single chain Fc fragment 4D5 were conjugated to mPA in sortase A-mediated reactions [99]. The obtained genetic fusions showed very similar cytotoxicities against HER2-positive breast cancer cell lines.

These examples demonstrated clearly the various options for fusing PA, and ideally mPA, to different ligands to retarget PA. It is important that the PA oligomerization is not affected and, therefore, fusions of other ligands have been made with the C-terminus of PA. The comparison of mPA-EGF with wild-type PA showed that this can be done without decreasing LFn delivery to the cytosol of target cells.

## 5. Conclusions

**Figure 2:**
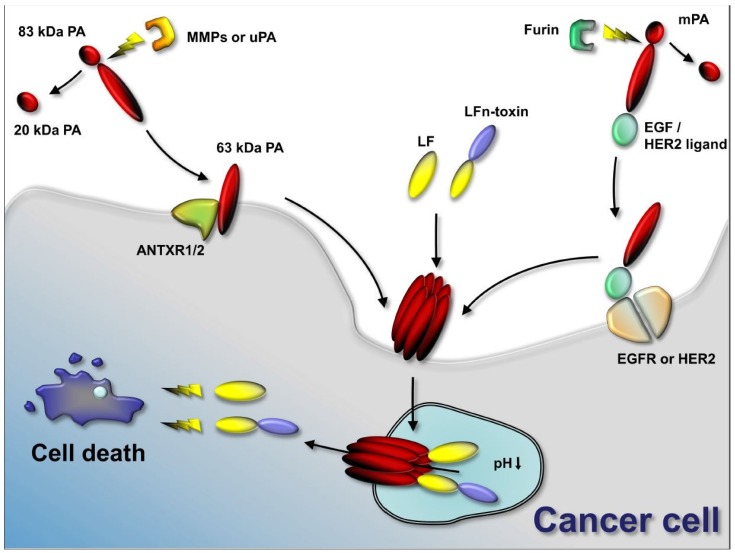
Anthrax toxin use in cancer therapies. Anthrax toxin component protective antigen (PA) can be mutated to contain restriction sites for tumor-selective proteases (such as matrix-metalloproteinases (MMPs) and urokinase plasminogen-activator (uPA)). In the tumor environment, MMPs or uPA cleave the mutated PA and the activated 63-kDa PA fragment binds its target receptors (ANTXR1 or ANTXR2) and assembles to oligomers. Anthrax toxin lethal factor (LF) or fusion proteins containing the *N*-terminus of LF and other protein toxins or enzymes bind to the PA oligomer. The complex is endocytosed and the low pH in the endosome results in pore formation by the PA oligomer. LF or LFn-toxin fusions unfold and translocate to the cytosol of the cancer cell. LF cleaves mitogen-activated protein kinase kinases and interferes with cellular signaling. Protein toxins delivered to the cancer cell interfere with protein synthesis, induce DNA damage and induce apoptosis. Alternatively to using mutated PA, PA can be retargeted to tumor-selective receptors, such as epidermal growth factor receptor (EGFR) or human epidermal growth factor receptor 2 (HER2) by genetic fusion of PA to EGF or HER2 ligands. Activation occurs in that case by furin and does not require specific tumor-selective proteases. Delivery of LF or LFn-toxin fusion occurs similarly.

This review describes, with many examples, the path to establishing AT as a versatile and powerful tool in biomedicine, and particularly in anti-tumor therapies (Figure 2). The focus of most studies lies on PA (or more specific PA variants) and LF or LFn genetic fusion proteins due to the more pronounced effect of LT on tumor growth. With many described modifications of PA to achieve more specific targeting to tumor cells, PA variants became the key factor for efficient targeting. The two main routes studied were either mutations in the protease cleavage site for PA activation and oligomerization or the complete re-targeting of PA by fusion with different ligands or antibody fragments. Many variants have been described for the two options and proved to be effective in every case.

Cellular delivery of LFn fusion proteins was described in detail in this review and still more examples are studied in various laboratories. The size of delivered fusion proteins varies from a few amino acids to proteins of more than 100 kDa. The cellular uptake is usually highly efficient and fast (within minutes to hours). Excellent understanding of the molecular mechanism of LF translocation helped to utilize the system for drug delivery. The main drawback of the mechanism might be the strict requirement for unfolding during translocation and refolding in the cytosol. Bulky effectors and highly stable proteins will not be delivered effectively—a limitation of the system, but one that may be amenable to the selection of protein variants with decreased stability.

The current studies on AT for tumor therapies rely on proteins produced in bacterial systems. The production can be performed in protease-reduced and non-infectious strains of Gram-positive *B. anthracis*. This system usually generates excellent protein yields with very low levels of lipopolysaccharides, thus facilitating preclinical testing in addition to mouse experiments. However, AT is a bacterial toxin and, thus, contains many immunogenic epitopes on its surface. An investigation to determine and mutate these antigens, as it has been done for PEIII, needs to be done in order to limit the immunogenicity of AT. Currently, combinations with drugs would be advisable to prevent the triggering of an immune response during a treatment phase. In conclusion, LT is a powerful protein delivery system with a well-characterized uptake mechanism. Past studies have unveiled many ways and methods for specific targeting of PA to different tumor types. Combination with potent effector molecules resulted in many successful mouse studies and future research should continue to build on the potential of AT for tumor therapies.

## Abbreviations

AT	anthrax toxin
Bcl-XL	B-cell lymphoma-extra large
CMG2 or ANTXR2	capillary morphogenesis gene 2
CdtB	cytolethal distending toxin B
DTA	diphtheria toxin A chain
EF	edema factor
ET	edema toxin
LF	lethal factor
LFn	LF *N*-terminal domain
LT	lethal toxin
MMPs	matrix-metalloproteases
MAPKKs	mitogen-activated protein kinase kinases
mPA	mutant PA
mPA-EGF	mPA genetically fused to human epidermal growth factor
mPA-ZHER2	mPA genetically fused to the affibody ZHER2
PA	protective antigen
PA-DK	PA with D512K mutation
PA-GN	PA with mutation complementing PA-D512K mutation
PE	*Pseudomonas* exotoxin A
PEIII	*Pseudomonas* exotoxin A catalytic domain
PE38	PE domains II and III
Raf-1	rapidly accelerated fibrosarcoma
TEM8 or ANTXR1	tumor endothelial marker 8
uPa	urokinase plasminogen activator
